# Meta-Analysis of Oral Anticoagulants and Adverse Outcomes in Atrial Fibrillation Patients After Intracranial Hemorrhage

**DOI:** 10.3389/fcvm.2022.961000

**Published:** 2022-07-15

**Authors:** Xin Liu, Siyu Guo, Zhicheng Xu

**Affiliations:** ^1^Department of Critical Care Medicine, The First Affiliated Hospital of Gannan Medical University, Ganzhou, China; ^2^Medical Department, Queen Mary School, Nanchang University, Nanchang, China; ^3^Department of Cardiology, Jiangxi Provincial People’s Hospital, The First Affiliated Hospital of Nanchang Medical College, Nanchang, China

**Keywords:** atrial fibrillation, intracranial hemorrhage, anticoagulation, prognosis, meta-analysis

## Abstract

**Background:**

Intracranial hemorrhage (ICH) is excluded in most anticoagulation randomized clinical trials (RCTs), so oral anticoagulant (OAC) therapy is still the conventional treatment for patients with atrial fibrillation (AF) after ICH. Therefore, we conducted a meta-analysis to determine the effectiveness and safety outcomes of OAC for these patients.

**Methods:**

We systematically searched the PubMed and Embase databases up to March 2022 for RCTs and observational studies exploring the effect of OAC in patients with AF after ICH. The effectiveness outcomes included stroke or systemic embolism, ischemic stroke, and all-cause death, whereas the safety outcomes were major bleeding and recurrent ICH. Hazard ratios (HRs) and 95% confidence intervals (CIs) from each study were pooled using a random-effects model.

**Results:**

A total of 14 studies were included. The OAC therapy that was performed reduced the risks of stroke or systemic embolism (HR = 0.65, 95% CI 0.53–0.81), ischemic stroke (HR = 0.70, 95% CI 0.60–0.82), and all-cause death (HR = 0.43, 95% CI 0.27–0.70) but had a higher risk of major bleeding (HR = 1.50, 95% CI 0.94–2.40) and showed no difference in recurrent ICH (HR = 0.91, 95% CI 0.53–1.55) compared to the no OAC therapy. With the use of non-vitamin K antagonist oral anticoagulant (NOAC) therapy, a lower risk of stroke or systemic embolism (HR = 0.83, 95% CI 0.70–0.98), all-cause death (HR = 0.67, 95% CI 0.53–0.84), and recurrent ICH (HR = 0.68, 95% CI 0.54–0.86) was observed against the use of vitamin K antagonists (VKA) therapy.

**Conclusion:**

The OAC therapy (especially VKA) revealed superior effectiveness in patients with AF after ICH, and the superiority of NOAC was also found, but some related evidence was limited.

## Introduction

Atrial fibrillation (AF) is a well-documented risk factor for stroke and systemic embolism (1, 2). The prevention of non-fatal and fatal thromboembolic events is a key goal for the management of patients with AF. Oral anticoagulants (OAC) are recommended in patients with AF to reduce the risk of stroke and thromboembolic events by national and international clinical practice guidelines (3). However, since intracranial hemorrhage (ICH) [especially symptomatic ICH (sICH)] is the most fatal complication of long-term anticoagulation (4), patients with previous ICH are regarded as an excluded population in the majority of randomized clinical trials (RCTs) of stroke prevention in AF. Hence, whether patients with AF after ICH derive net clinical benefit (including efficacy and safety outcomes) from antithrombotic therapy is still unclear, given that the effect of ischemic stroke reduction is needed to balance against increased bleeding recurrence in this population. A previous meta-analysis by Korompoki et al., which pooled seven observational studies and 2,452 ICH survivors with AF, demonstrated that anticoagulation with vitamin K antagonists (VKA) correlated with a lower rate of ischemic stroke and no significantly increased ICH recurrence, as compared with antiplatelet agents or no antithrombotic medication (5, 6). Nevertheless, because of the limited high-grade evidence in this specific population (7), whether to use anticoagulation therapy and the specific therapy window for patients with AF after ICH is still inconclusive.

Although OAC including the non-vitamin K antagonist oral anticoagulants (NOAC; i.e., factor Xa inhibitors and direct thrombin inhibitor) and warfarin are all effective in preventing AF-related stroke, NOAC has been shown to correlate with a significantly lower risk of ICH than VKA in patients with AF without prior ICH (8). Moreover, our recent meta-analysis of 17 retrospective cohort studies found that apixaban was superior to dabigatran or rivaroxaban in stroke prevention with lower bleeding risk in patients with AF (9). However, in the clinical trials performed by Schreuder et al., the apixaban allocated group elaborated the annual risk of non-fatal stroke or vascular death and a higher risk of major bleeding compared with the no anticoagulation treated group (10). Moreover, by analyzing the result of Lewis et al., the OAC-treated group demonstrated lower rates of recurrent ICH than the no OAC group, but the level of evidence was relatively weak to draw this explicit conclusion (11). Therefore, we aimed to investigate the effectiveness and safety of OAC (NOAC and VKA) compared with no OAC and evaluated the effect of the NOAC therapy versus the VKA therapy in patients with AF after ICH.

## Methods

We conducted this meta-analysis based on the criteria of the Cochrane Handbook for Systematic Reviews of Interventions (version 6.2). The results were presented according to the Preferred Reporting Items for Systematic review and Meta-Analyses (PRISMA) 2020 statement (Supplementary Table 1; 12).

### Search Strategy

Two reviewers performed the literature search, systematically searching the PubMed and Embase database sources up to March 2022 for studies exploring the effect of OAC compared with no OAC in patients with AF after ICH. The following search terms were used: (1) “AF” OR “atrial flutter,” (2) “ICH” OR “intracranial bleeding” OR “intracerebral hemorrhage” OR “hemorrhagic stroke” OR “ICH,” (3) “OAC” OR “vitamin K antagonist” OR “VKA” OR “warfarin” OR “non-vitamin K antagonist oral anticoagulant” OR “direct oral anticoagulant” OR “novel oral anticoagulant” OR “NOAC” OR “DOAC” OR “dabigatran” OR “rivaroxaban” OR “apixaban” OR “edoxaban.” The aforementioned three categories of search terms were combined using the Boolean operator “and.” The detailed search strategies are shown in Supplementary Table 2. In addition, the reference lists of the retrieved articles and prior reviews were manually checked for additional eligible studies.

### Inclusion and Exclusion Criteria

Randomized clinical trials or observational (prospective or retrospective cohort) studies were included if they focused on at least one of the effectiveness and safety outcomes of OAC compared with no OAC in non-valvular AF patients after ICH. The OAC included VKA or NOAC, whereas those in the reference were patients with antiplatelet or no antithrombotic agents. Since the pooled analysis could be performed for the outcome that was simultaneously reported in at least two included studies, we chose the effective outcomes including stroke or systemic embolism, ischemic stroke, and all-cause death, and the safety outcomes, including major bleeding and recurrent ICH. Based on the definition of major bleeding, according to the International Society on Thrombosis and Hemostasis criteria, gastrointestinal, genitourinary, respiratory tract, ICH/sICH, and other fatal and symptomatic bleeding in critical organs were regarded as severe hemorrhagic events for hospitalization. The definitions of these outcomes were applied according to the originally included studies. For the observational studies, the confounders were adjusted *via* the propensity score methods (e.g., matching, inverse probability of treatment weighting) or the regression model adjustment. The effects of OAC on the studied outcomes were expressed as adjusted hazard ratios (HRs) and 95% confidence intervals (CIs).

We excluded the studies focusing on AF patients with non-ICH bleeding (e.g., any bleeding, gastrointestinal bleeding, major bleeding, and microbleed) or patients with cardioversion, ablation, or left atrial appendage (LAA). The studies without adjustment or with a sample size of <100 were excluded, due to limited convincing evidence being provided. In addition, we also excluded certain publication types (e.g., reviews, comments, case reports, case series, letters, editorials, and meeting abstracts) due to insufficient data or study details. If there were overlapping data among two or more studies, we included the one with the largest sample size or the longest follow-up duration.

### Study Selection and Data Abstraction

Two reviewers independently screened the titles and abstracts of the retrieved studies from the electronic databases. Subsequently, based on the pre-defined inclusion criteria, we selected the eligible studies after the full-text screenings. Disagreements were resolved through discussion between the two reviewers or after consulting with the corresponding authors. The following data of the included studies were abstracted: study characteristics (first author, year of publication, data source, study period, and study design), study population, and baseline characteristics (age, male ratio, sample size, stroke and bleeding risk prediction scores, and drugs in the OAC group), effectiveness and safety outcomes, follow-up period, and outcome data (sample size and the number of events between groups, and adjusted HRs). For those studies reporting adjusted data with multiple models, we applied the most adjusted one.

### Study Quality Assessment

The bias risks of RCTs were assessed using Cochrane’s Risk of Bias tool, which mainly included six domains: selection bias, performance bias, detection bias, attrition bias, reporting bias, and other risk biases. The level of the bias risk in each domain was scored as “low,” “unclear,” or “high” risk. In addition, the Newcastle-Ottawa Scale (NOS) tool was used to assess the quality of observational cohort studies. In this meta-analysis, the NOS of ≥6 and <6 points were scored as moderate-to-high quality and low quality, respectively, (9, 13).

### Statistical Analysis

All the statistical analyses of this meta-analysis were conducted using Review Manager version 5.4 software (the Cochrane Collaboration 2014, Nordic Cochrane Centre Copenhagen, Denmark)^1^.

The statistical heterogeneity across the included studies was assessed using the *p*-value of the Cochrane *Q* test and the *I*^2^ statistic, where a *p*-value of <0.10 in the Cochrane *Q* test or an *I*^2^ value of >50% suggested significant heterogeneity. We excluded the included studies one by one to find out the potential source of high heterogeneity. In the pooled analysis, the effectiveness and safety outcomes in patients with AF after ICH were examined among three comparisons, namely OAC versus no OAC, VKA versus no VKA, and NOAC versus VKA. The adjusted HRs and 95% CIs were converted to the natural logarithms [Ln (HR)] and their corresponding standard errors [Ln (upper CI)-Ln (lower CI)/3.92], which were pooled by a DerSimonian and Laird random-effects model with an inverse variance method. The subgroup analysis and sensitivity analysis were not conducted due to the limiting included studies. The publication bias for the reported effect estimates was assessed using the funnel plots in which the logHRs were plotted against their standard errors. In addition, Egger’s and Begg’s tests for each outcome were applied to examine the statistical publication bias.

## Results

### Study Selection

**FIGURE 1:**
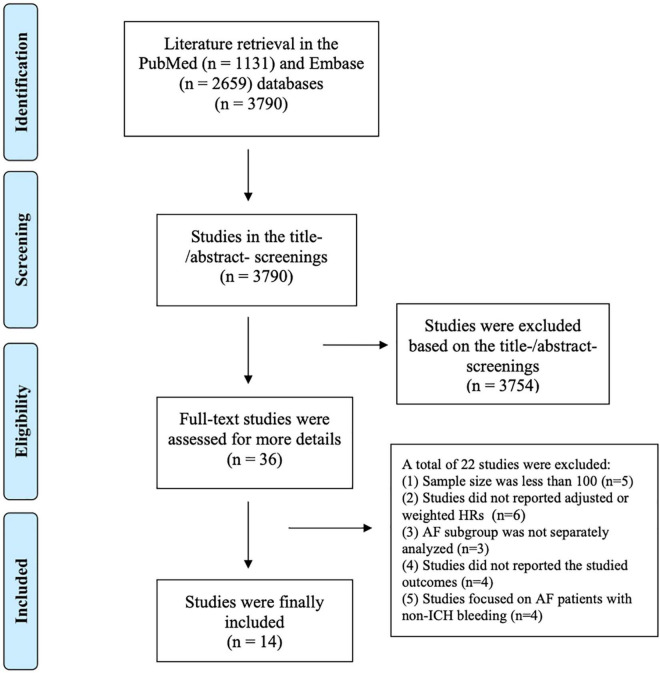
The flow chart of literature retrieval of this meta-analysis.

The flow chart of literature retrieval is shown in Figure 1. A total of 3,790 records were retrieved in the two databases of PubMed and Embase; after the first phase of the title and abstract screenings, 36 remaining studies were potentially suitable and further assessed by full-text screenings. According to the pre-defined inclusion and exclusion criteria, we subsequently excluded 22 studies because (1) the sample size was less than 100 (*n* = 5); (2) the studies did not report adjusted or weighted HRs (*n* = 6); (3) the studies focused on a mixed population, and the AF subgroup was not separately analyzed (*n* = 3); (4) the studies did not report the studied outcomes (*n* = 4); and (5) the studies focused on AF patients with non-ICH bleeding (*n* = 4; Supplementary Table 3). Finally, a total of 14 studies (2 RCTs and 12 observational cohorts; 10, 11, 14–25) were included in our meta-analysis.

Baseline characteristics of the included studies are presented in Table 1. Among the included studies, three are from Denmark, two from Korea, three from Taiwan, and one each from the United Kingdom, Netherlands, Sweden, United States, Canada, and Germany. The mean age of patients ranged from 68.5 to 83.0 years, with a sample size between 101 and 12,917. Evaluated CHA2DS2-VASc, congestive heart failure/left ventricular ejection fraction ≤40%, hypertension, age ≥ 75 years (2 points), diabetes mellitus, prior stroke/transient ischemic attack/thromboembolism (2 points), vascular disease, age 65-74 years, female sex; HAS-BLED, Hypertension, Abnormal liver/renal function, Stroke, Bleeding history or predisposition, Labile international normalized ratio, Elderly, Drugs/alcohol concomitantly scores ranged from 3.26 to 6.0, and 2.0 to 4.4, respectively. Data on a specific classification of ICH were not available in seven studies (Supplementary Table 4). The adjusted risk factors in each included study are shown in Supplementary Table 5.

**TABLE 1:** Baseline characteristics of the included studies in this meta-analysis.

Studies	Database source	Inclusion period	Study design	Study population	Age (y)	Males (%)	Sample size (N)	CHA 2DS2-VASc	HAS-BLED	OAC group	No OAC group	Follow-up	Time to prescription of OACs
Lewis	SoSTART; United Kingdom	2018–2020	RCT	AF patients who had survived at least 24 h after symptomatic spontaneous ICH	79.0	63	203	4.0	2.0	DOACs (dabigatran apixaban, rivaroxaban, edoxaban) or VKAs (warfarin, acenocoumarol, phenindione)	Antiplatelets* or no antithrombotic agents	1.2 year	24 h
Schreuder et al. (10)	APACHE-AF; Netherlands	2015–2016	RCT	Patients with a spontaneous ICH in the prior 7–90 days during anticoagulation for AF	78.0	54	101	4.0	NA	DOACs (apixaban)	Antiplatelets or no antithrombotic agents	1.9 year	45 (22–70) days
Komen et al. (14)	The Stockholm Healthcare Database; Sweden	2011–2018	Observational cohort	AF patients who were diagnosed with ICH	80.2	NA	3,006	NA	NA	DOACs (dabigatran apixaban, rivaroxaban, edoxaban) or VKAs (Warfarin)	No anticoagulants and no antiplatelets	90 day	NA
Lee et al. (15)	The Korean Health Insurance Review and Assessment database; South Korea	2010–2018	Observational cohort	Asian patients with AF and a history of ICH	72.4	56.9	5,712	4.0	4.4	DOACs (dabigatran apixaban, rivaroxaban, edoxaban) or VKAs (Warfarin)	None	9.27 year	3.1 ± 2.8 (years)
Tsai et al. (16)	The National Health Insurance Research Database; Taiwan	2012–2016	Observational cohort	Asian patients with AF and a history of ICH	76.0	58.4	4,540	5.55	4.31	DOACs (dabigatran apixaban, rivaroxaban) or VKAs (Warfarin)	None	5.0 year	NA
Newman et al. (17)	Medicare Part D Claims Data; United States	2010–2016	Observational cohort	AF who experienced an OAC-related ICH and survived at least 6 weeks after the ICH	NA	43.7	1,502	NA	NA	DOACs (dabigatran apixaban, rivaroxaban) or VKAs (Warfarin)	Antiplatelets or no antithrombotic agents	780 day	6 weeks
Nielsen et al. (18)	Danish nationwide databases; Denmark	2003–2017	Observational cohort	AF patients sustaining an ICH and who subsequently claimed an OAC prescription	76.1	60.9	622	4.4	NA	DOACs (dabigatran apixaban, rivaroxaban) or VKAs (Warfarin)	None	3.0 year	2 months
Perreault et al. (19)	The Quebec Régie de l’Assurance Maladie du Québec and Med-Echo administrative databases; Canada	1995–2015	Observational cohort	AF patients with an incident ICH requiring admission to a hospital	83.0	46.9	683	3.9	2.6	DOACs or VKAs	No anticoagulants and no antiplatelets	1.0 year	6 weeks
Nielsen et al. (20)	Danish nationwide databases; Denmark	1998–2016	Observational cohort	AF patients sustaining an ICH (hemorrhagic stroke or traumatic ICH) and who subsequently claimed an OAC prescription	77.1	61.3	2,415	3.9	3.6	VKAs (Warfarin)	Antiplatelets or no antithrombotic agents	1.0 year	10 weeks
Chao et al. (21)	The National Health Insurance Research Database; Taiwan	1996–2011	Observational cohort	Asian patients with AF and a history of ICH	74.7	57.0	12,917	6.0	NA	VKAs (Warfarin)	No anticoagulants and no antiplatelets	3.3 year	30 days
Park et al. (22)	The Institutional Review Board of Severance Cardiovascular Hospital, Seoul; South Korea	2009–2013	Observational cohort	Patients with AF and a history of ICH	68.5	34.1	428	3.26	3.48	VKAs (Warfarin)	Antiplatelets or no antithrombotic agents	39.5 m	117.5 ± 235.7 (days)
Nielsen et al. (23)	Danish nationwide databases; Denmark	1997–2013	Observational cohort	AF patients sustaining an ICH and who subsequently claimed an OAC prescription	78.0	62.0	1,752	3.9	3.2	DOACs (dabigatran apixaban, rivaroxaban, edoxaban) or VKAs (coumarin)	No anticoagulants and no antiplatelets	1.0 year	6 months
Kuramatsu et al. (24)	19 German tertiary care centers; Germany	2006–2012	Observational cohort	AF patients had OAC-associated ICH^※^	75.0	61.0	566	NA	NA	VKAs (Warfarin)	Antiplatelets or no antithrombotic agents	1.0 year	95 (44–180) minutes
Lin et al. (25)	Health and Welfare Database; Taiwan	2011–2017	Observational cohort	Asian patients with AF and a history of ICH	76.4	58.7	2,640	5.1	NA	DOACs or VKAs (Warfarin)	Antiplatelets or no antithrombotic agents	0.6 year	42 (10–127) days

*Aspirin and/or P2Y12 antagonist treatment. ^※^ICH patients with AF were only a part of the whole population in the included study. ^#^only used in the subgroup analysis of VKAs versus no VKAs. AF, atrial fibrillation; ICH, intracranial hemorrhage; OAC, oral anticoagulation; DOACs, direct oral anticoagulants; VKAs, vitamin K antagonists; RCT, randomized controlled Trial; SoSTART, the start or stop anticoagulants randomized trial; APACHE-AF, the apixaban versus antiplatelet drugs or no antithrombotic drugs after anticoagulation-associated intracerebral hemorrhage in patients with atrial fibrillation; CHA2DS2-VASc, congestive heart failure/left ventricular ejection fraction ≤ 40%, hypertension, age ≥ 75 years (2 points), diabetes mellitus, prior stroke/transient ischemic attack/thromboembolism (2 points), vascular disease, age 65–74 years, female sex; HAS-BLED, Hypertension, Abnormal liver/renal function, Stroke, Bleeding history or predisposition, Labile international normalized ratio, Elderly, Drugs/alcohol concomitantly; and NA, not available.

### Risk of Bias Within Studies

The risk of bias assessment for RCT is presented in Supplementary Table 6, and quality assessment for observational cohorts is shown in Supplementary Table 7. These two assessments revealed that these 12 included observational cohorts and two RCTs’ quality were relatively high and the result is convincing. Schreuder et al. showed a high risk of selection and performance bias lacking blindness in participants, their treating physicians, and local investigators.

### Effect of Oral Anticoagulants Versus no Oral Anticoagulants in Atrial Fibrillation Patients After Intracranial Hemorrhage

**FIGURE 2:**
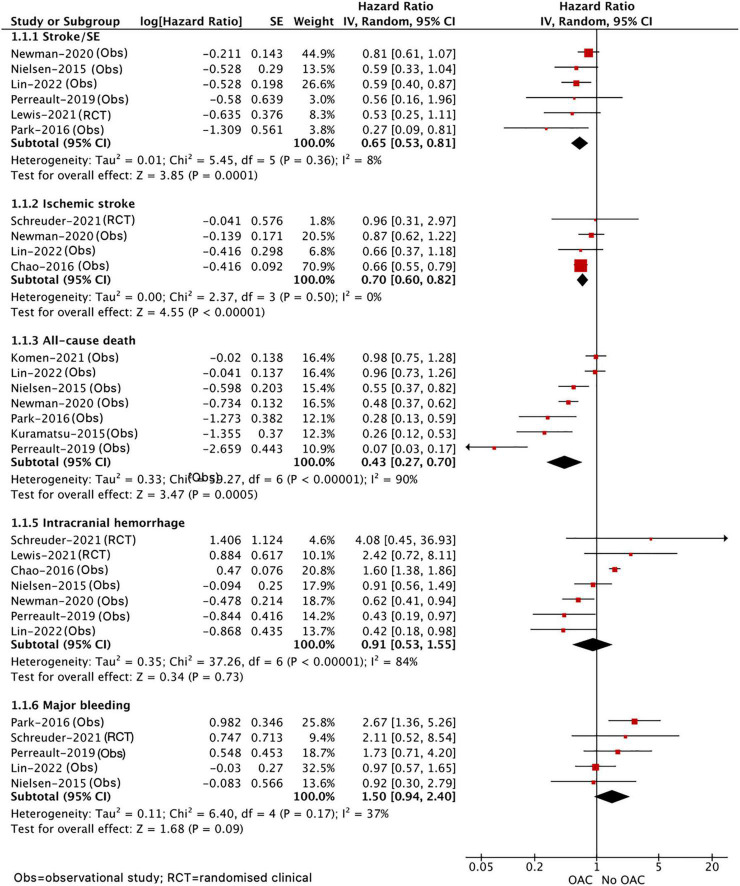
Comparing the efficacy of OAC with no OAC in patients with AF after ICH. AF, atrial fibrillation; ICH, intracranial hemorrhage; OAC, oral anticoagulants; CI, confidence interval; IV, the inverse of the variance; SE, standard error; and SE, systemic embolism.

As shown in Figure 2, our pooled results based on the random-effects model showed that, compared with no OAC, the use of OAC (NOAC or VKA) was significantly correlated with reduced risks of effectiveness outcomes including stroke or systemic embolism (HR = 0.65, 95% CI 0.53–0.81; *I*^2^ = 8%), ischemic stroke (HR = 0.70, 95% CI 0.60–0.82; *I*^2^ = 0%), and all-cause death (HR = 0.43, 95% CI 0.27–0.70; *I*^2^ = 90%) and showed an upward trend toward major bleeding (HR = 1.50, 95% CI 0.94–2.40; *I*^2^ = 37%) but showed no difference in recurrent ICH (HR = 0.91, 95% CI 0.53–1.55; *I*^2^ = 84%) between the two studied groups. Although we failed to find the source of high heterogeneity, the results were stable when excluding each included study at a time.

**FIGURE 3:**
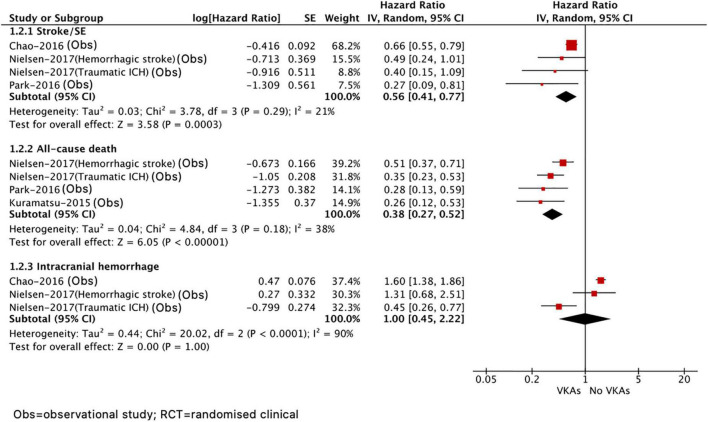
Comparing the efficacy of VKAs with no VKAs in patients with AF after ICH. AF, atrial fibrillation; ICH, intracranial hemorrhage; VKAs, vitamin K anticoagulants; CI, confidence interval; IV, the inverse of the variance; and SE, standard error.

As presented in Figure 3, compared with no VKA, the use of VKA was correlated with decreased risks of stroke or systemic embolism (HR = 0.56, 95% CI 0.41–0.77; *I*^2^ = 21%) and all-cause death (HR = 0.38, 95% CI 0.27–0.52; *I*^2^ = 38%). There was no difference in recurrent ICH (HR = 1.00, 95% CI 0.45–2.22, *I*^2^ = 90%) between VKA versus no VKA; however, this should be interpreted cautiously due to a quite wide CI and significant heterogeneity. In addition, we did not assess the effect of NOAC versus no NOAC in patients with AF after ICH because only the included study by Komen et al. (14) reported this comparison.

### Effect of Non-vitamin K Antagonist Oral Anticoagulants Versus Vitamin K Antagonists in Atrial Fibrillation Patients After Intracranial Hemorrhage

**FIGURE 4:**
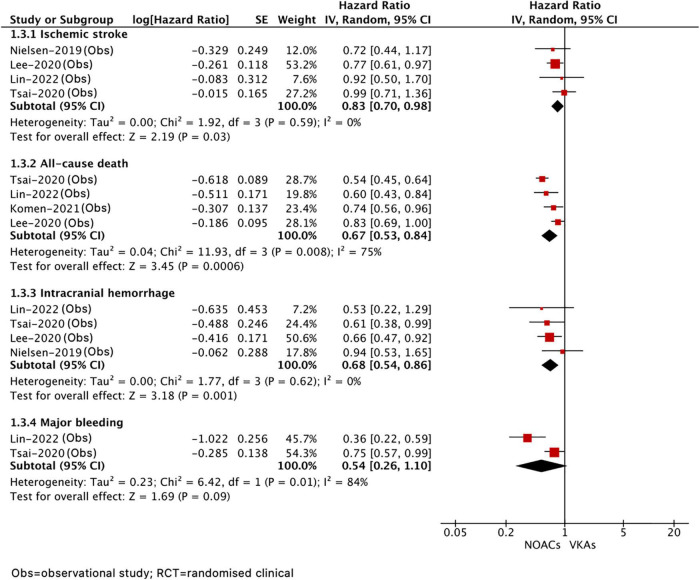
Comparing the efficacy of NOACs with VKAs in patients with AF after ICH. AF, atrial fibrillation; ICH, intracranial hemorrhage; VKAs, vitamin K anticoagulants; NOACs, non-vitamin K oral anticoagulants; CI, confidence interval; IV, the inverse of the variance; and SE, standard error.

A total of five included studies reported the effects of NOAC versus VKA in patients with AF after ICH. As shown in Figure 4, our results based on the random-effects model showed that, compared with VKA, the use of NOAC was significantly correlated with reduced risks of stroke or systemic embolism (HR = 0.83, 95% CI 0.70–0.98; *I*^2^ = 0%), all-cause death (HR = 0.67, 95% CI 0.53–0.84; *I*^2^ = 75%), and recurrent ICH (HR = 0.68, 95% CI 0.54–0.86, *I*^2^ = 0%), but there was no significant difference in major bleeding (HR = 0.54, 95% CI 0.26–1.10, *I*^2^ = 84%).

### Publication Bias

As shown in Supplementary Figures 1–3, we observed no potential publication biases for the effectiveness and safety outcomes by assessing the funnel plots. Egger’s and Begg’s tests also suggest no publication biases for most outcomes (*p* > 0.1; Supplementary Table 8).

## Discussion

The main findings of our meta-analysis on the effectiveness and safety outcomes of OAC versus no OAC in patients with AF after ICH are summarized as follows: (1) OAC was correlated with a lower risk of stroke or systemic embolism, ischemic stroke, and all-cause death but a similar risk of major bleeding and major recurrent ICH compared with no OAC; (2) VKA treatment had significantly reduced risks of stroke or systemic embolism and all-cause death but a similar risk of recurrent ICH compared with no VKA treatment; (3) NOAC had better effectiveness and safety outcomes than VKA, demonstrating a significant reduction of ischemic stroke, all-cause death, and lower risk of recurrent ICH, but no significant difference in major bleeding. Although these results should be interpreted cautiously because of limited evidence, VKA might not be the preferred option because of their higher risk of recurrent ICH than NOAC in patients with AF after ICH. Our results may provide valuable evidence for the current uncertain management of stroke prevention for AF patients with ICH (26).

Based on hematoma-mediated inflammation, antithrombotic drug interruption, and common vascular risk factors, survivors of ICH are at a higher risk of ischemic stroke compared with the general population (27, 28); therefore, stroke prevention is crucial for this specific population. Previous observational studies have provided evidence in favor of recommencing anticoagulation therapy. Several prior studies showed that anticoagulation was correlated with reduced risks of ischemic stroke or systemic embolism and all-cause death (20–22). A recent observational study by Newman et al., which enrolled 1,502 OAC-related ICH survivors, demonstrated that anticoagulation was correlated with a lower risk of ICH (17). In our analysis, after integrating all the available data, it was found that OAC-treated patients had a reduced risk of stroke or systemic embolism, all-cause death and also an upward trend toward major bleeding as compared with no anticoagulants. Given that VKA and NOAC are both effective anticoagulants for the prevention of thromboembolic events in patients with AF, it is logical to observe a favorable effectiveness profile of OAC treatment in patients with AF after ICH. In the present study, no significant difference in recurrent ICH risk was also observed between patients with and without OAC treatment, which was consistent with those reported by recent RCTs in patients with AF after ICH (10, 11). These safety outcomes suggest that OAC therapy may not serve as a promoter for secondary hemorrhagic stroke occurrence. Compared with those with no OAC therapy, the VKA-treated group demonstrates superior effectiveness of anticoagulation and no significant difference in recurrent ICH, which is consistent with the result of the OAC group versus no OAC group. However, due to only one related study, we did not perform the analysis of the NOAC group versus the no NOAC group to evaluate the effectiveness and safety profiles of the NOAC therapy in patients with AF after ICH. In addition, because the sample size of these two recent RCTs is relatively small, further research should be warranted to provide highly correlated evidence for identifying the association between anticoagulant treatment and ICH prevention in patients with AF after ICH.

Considering the increased risk of bleeding with anticoagulants, a treatment strategy that provides a better safety profile may be the optimal option for patients with AF after ICH. Focusing on the east Asian population with cardiovascular diseases, patients who were prescribed antithrombotic agents were predisposed to bleeding events, such as gastrointestinal bleeding and ICH, etc. (29). Data from the present studies demonstrated superior effectiveness and safety outcomes of NOAC in stroke or systemic embolism and recurrent ICH compared with VKA, which was consistent with associated RCTs and observational cohorts in AF patients with previously diagnosed ICH (11, 23–25). A prior meta-analysis, which pooled 48 randomized trials and 71,683 patients with AF, suggested that the superior effectiveness and safety profiles of NOAC were attributed to the prevention of stroke, which included ICH and ischemic stroke (8). Moreover, a previous observational study by Hagii et al. elaborated that the size of hematoma in NOAC-associated ICH was smaller than that of VKA-associated ICH (30). Consequently, by reducing the incidence of hemorrhagic stroke, NOAC was significantly correlated with a lower risk of recurrent ICH, which was a crucial safety outcome in the risk-benefit assessment of anticoagulant therapy in patients with AF (31–34). In the current stage, at least 4 pivotal randomized trials (RE-LY, ROCKET AF, ARISTOTLE, and ENGAGE AF-TIMI 48) confirm the superior safety outcomes (especially for lower bleeding risk) of NOAC versus VKA, and our results are consistent with this significant finding.

In addition, NOAC treatment confers benefits for survival in patients with AF after ICH as compared with VKA treatment. This beneficial effect is correlated with the prevention of stroke or systemic embolism and the reduction of recurrent ICH in NOAC treatment. Our findings are consistent with that of a previously published meta-analysis, with almost 50% lower risk of ICH in AF patients without previous ICH, though no obvious difference in OAC-ICH was found between the use of NOAC and VKA (6, 8). It reveals that NOAC may be recommended to VKA-tolerant patients with AF after ICH to prevent ICH recurrence. Nevertheless, further studies focusing on specific NOAC subgroups should be performed to analyze consequent all-cause death. Our meta-analysis provides a future research orientation to analyze the discrepancies among secondary outcomes of different NOAC subgroups. Besides anticoagulant treatment, another available and radical approach for AF patients after ICH management is LAA occlusion. In several national observational studies and RCTs (PROTECT AF and PREVAIL), it was demonstrated that, compared with VKA treatment, the LAA occlusion was non-inferior to preventing stroke and major bleeding (35–37). The LAA occlusion is a potential alternative approach for patients with AF who are contraindicated to anticoagulant treatment. However, the limited RCTs comparing anticoagulant treatment (especially for NOAC therapy) with LAA occlusion are performed to identify the effectiveness and safety outcomes of LAA occlusion. There are two ongoing RCTs: CLEARANCE (NCT04298723), assessing LAA occlusion plus a short-term anticoagulant therapy versus anticoagulant treatment in 550 AF patients with ICH; STROKECLOSE (NCT02830152), a study of LAA occlusion versus other medical therapies (NOAC, VKA, antiplatelet therapy, and no antithrombotic therapy at all) in 750 AF patients after ICH. Further associated research should be performed to provide more convincing evidence.

## Limitation

Our study had several limitations. First, we pooled data from mostly observational studies and two recent RCTs with a limited sample size, which might limit the data quality. Second, the severity, imaging, and dysfunction of prior stroke/transient ischemic attack or ICH were not addressed and adjusted, which may bring heterogeneity to the pooled result. For example, for patients with a high risk of ICH recurrence, clinical physicians may tend to not prescribe VKA. In addition, the different criteria for major bleeding definition exist in our involving research. Third, due to limited data sources of comparison between OAC and no OAC treatment in AF patients with previous ICH, our study did not divide the patients into four subgroups with traumatic, OAC-related, spontaneous, and no classification ICH for subgroup analysis. Fourth, the limited data were not available for evaluating the effectiveness and safety outcomes among different NOAC strategies and NOAC versus no NOAC. Further research is warranted to examine the outcomes of dabigatran, apixaban, rivaroxaban, and edoxaban in patients with AF after ICH, respectively. Moreover, due to the diverse anticoagulation commencement time presented by our 14 included studies, early or late anticoagulation strategy may have a different effect on ischemic stroke, major bleeding, ICH, and other outcomes, but we did not perform a subgroup analysis to stratify patients into distinct OAC prescription time. Finally, the volume of hemorrhage also plays an important role in anticoagulation decisions, but we were unable to perform the related subgroup analysis.

## Conclusion

Oral anticoagulant treatment exhibited superior effectiveness profiles in patients with AF after ICH, without increasing the risk of recurrent ICH and major bleeding. Especially, NOAC exerted more favorable effects on stroke prevention and mortality with a lower risk of recurrent ICH as compared with VKA. However, due to insufficient evidence provided by limited RCTs, further research should be performed to identify the superiority of NOAC.

## Data Availability Statement

The original contributions presented in this study are included in the article/Supplementary Material, further inquiries can be directed to the corresponding authors.

## Author Contributions

All authors listed have made a substantial, direct, and intellectual contribution to the work, and approved it for publication.

## Conflict of Interest

The authors declare that the research was conducted in the absence of any commercial or financial relationships that could be construed as a potential conflict of interest.

## Publisher’s Note

All claims expressed in this article are solely those of the authors and do not necessarily represent those of their affiliated organizations, or those of the publisher, the editors and the reviewers. Any product that may be evaluated in this article, or claim that may be made by its manufacturer, is not guaranteed or endorsed by the publisher.

## Footnotes

1
https://community.cochrane.org/
