# Supplementary material for: Meta-Analysis of Oral Anticoagulants and Adverse Outcomes in Atrial Fibrillation Patients After Intracranial Hemorrhage
Source: Front Cardiovasc Med. 2022 Jul 15;9:961000. doi: 10.3389/fcvm.2022.961000 (PMC9334654; doi:10.3389/fcvm.2022.961000)
Supplement: Supplementary file 1 [file Data_Sheet_1.PDF]

Supplemental Table 1 PRISMA 2020 Checklist

Section and Topic	Item #	Checklist item	Location where item is reported
**TITLE**			
Title	1	Identify the report as a systematic review.	Page 1
**ABSTRACT**			
Abstract	2	See the PRISMA 2020 for Abstracts checklist.	Page 2
**INTRODUCTION**			
Rationale	3	Describe the rationale for the review in the context of existing knowledge.	Page 4
Objectives	4	Provide an explicit statement of the objective(s) or question(s) the review addresses.	Page 4, 5
**METHODS**			
Eligibility criteria	5	Specify the inclusion and exclusion criteria for the review and how studies were grouped for the syntheses.	Page 5, 6
Information sources	6	Specify all databases, registers, websites, organisations, reference lists and other sources searched or consulted to identify studies. Specify the date when each source was last searched or consulted.	Page 5
Search strategy	7	Present the full search strategies for all databases, registers and websites, including any filters and limits used.	Page 5
Selection process	8	Specify the methods used to decide whether a study met the inclusion criteria of the review, including how many reviewers screened each record and each report retrieved, whether they worked independently, and if applicable, details of automation tools used in the process.	Page 6, 7
Data collection process	9	Specify the methods used to collect data from reports, including how many reviewers collected data from each report, whether they worked independently, any processes for obtaining or confirming data from study investigators, and if applicable, details of automation tools used in the process.	Page 6, 7
Data items	10a	List and define all outcomes for which data were sought. Specify whether all results that were compatible with each outcome domain in each study were sought (e.g. for all measures, time points, analyses), and if not, the methods used to decide which results to collect.	Page 6, 7

Section and Topic	Item #	Checklist item	Location where item is reported
Data items	10b	List and define all other variables for which data were sought (e.g. participant and intervention characteristics, funding sources). Describe any assumptions made about any missing or unclear information.	Page 6, 7
Study risk of bias assessment	11	Specify the methods used to assess risk of bias in the included studies, including details of the tool(s) used, how many reviewers assessed each study and whether they worked independently, and if applicable, details of automation tools used in the process.	Page 7
Effect measures	12	Specify for each outcome the effect measure(s) (e.g. risk ratio, mean difference) used in the synthesis or presentation of results.	Page 7
Synthesis methods	13a	Describe the processes used to decide which studies were eligible for each synthesis (e.g. tabulating the study intervention characteristics and comparing against the planned groups for each synthesis (item #5)).	Page 7, 8
	13b	Describe any methods required to prepare the data for presentation or synthesis, such as handling of missing summary statistics, or data conversions.	Page 7, 8
	13c	Describe any methods used to tabulate or visually display results of individual studies and syntheses.	Page 7, 8
	13d	Describe any methods used to synthesize results and provide a rationale for the choice(s). If meta-analysis was performed, describe the model(s), method(s) to identify the presence and extent of statistical heterogeneity, and software package(s) used.	Page 7, 8
	13e	Describe any methods used to explore possible causes of heterogeneity among study results (e.g. subgroup analysis, meta-regression).	Page 7, 8
	13f	Describe any sensitivity analyses conducted to assess robustness of the synthesized results.	Page 7, 8
Reporting bias assessment	14	Describe any methods used to assess risk of bias due to missing results in a synthesis (arising from reporting biases).	Page 7, 8

Section and Topic	Item #	Checklist item	Location where item is reported
Certainty assessment	15	Describe any methods used to assess certainty (or confidence) in the body of evidence for an outcome.	Page 7, 8
**RESULTS**			
Study selection	16a	Describe the results of the search and selection process, from the number of records identified in the search to the number of studies included in the review, ideally using a flow diagram.	Page 8
	16b	Cite studies that might appear to meet the inclusion criteria, but which were excluded, and explain why they were excluded.	Page 8
Study characteristic s	17	Cite each included study and present its characteristics.	Page 8, 9
Risk of bias in studies	18	Present assessments of risk of bias for each included study.	Page 9
Results of individual studies	19	For all outcomes, present, for each study: (a) summary statistics for each group (where appropriate) and (b) an effect estimate and its precision (e.g. confidence/credible interval), ideally using structured tables or plots.	Page 9, 10
Results of syntheses	20a	For each synthesis, briefly summarise the characteristics and risk of bias among contributing studies.	Page 9, 10
	20b	Present results of all statistical syntheses conducted. If meta-analysis was done, present for each the summary estimate and its precision (e.g. confidence/credible interval) and measures of statistical heterogeneity. If comparing groups, describe the direction of the effect.	Page 9, 10
	20c	Present results of all investigations of possible causes of heterogeneity among study results.	Page 9, 10
	20d	Present results of all sensitivity analyses conducted to assess the robustness of the synthesized results.	Page 9, 10

Section and Topic	Item #	Checklist item	Location where item is reported
Reporting biases	21	Present assessments of risk of bias due to missing results (arising from reporting biases) for each synthesis assessed.	Page 10
Certainty of evidence	22	Present assessments of certainty (or confidence) in the body of evidence for each outcome assessed.	Page 10
**DISCUSSION**			
Discussion	23a	Provide a general interpretation of the results in the context of other evidence.	Page 10-13
	23b	Discuss any limitations of the evidence included in the review.	Page 13
	23c	Discuss any limitations of the review processes used.	Page 13
	23d	Discuss implications of the results for practice, policy, and future research.	Page 11-13
**OTHER INFORMATION**			
Registration and protocol	24a	Provide registration information for the review, including register name and registration number, or state that the review was not registered.	Page 5
	24b	Indicate where the review protocol can be accessed, or state that a protocol was not prepared.	Page 5
	24c	Describe and explain any amendments to information provided at registration or in the protocol.	Page 5
Support	25	Describe sources of financial or non-financial support for the review, and the role of the funders or sponsors in the review.	Page 14
Competing interests	26	Declare any competing interests of review authors.	Page 14
Availability of data, code and other materials	27	Report which of the following are publicly available and where they can be found: template data collection forms; data extracted from included studies; data used for all analyses; analytic code; any other materials used in the review.	In Supplemental table and figure

From: Page MJ, McKenzie JE, Bossuyt PM, Boutron I, Hoffmann TC, Mulrow CD, et al. The PRISMA 2020 statement: an updated guideline for reporting systematic reviews. BMJ 2021;372:n71. doi: 10.1136/bmj.n71

For more information, visit: http://www.prisma-statement.org/

Supplemental Table 2 The search strategies until March 1, 2022

	Search terms	Items
**PubMed**		
#1	“atrial fibrillation” OR “atrial flutter”	100,096
#2	“intracranial hemorrhage” OR “intracranial bleeding” OR “intracerebral hemorrhage” OR “hemorrhagic stroke” OR “ICH”	36,958
#3	“oral anticoagulant” OR “vitamin K antagonist” OR “VKA” OR ‘warfarin’ OR “non-vitamin K antagonist oral anticoagulant” OR “direct oral anticoagulant” OR “novel oral anticoagulant” OR “NOAC” OR “DOAC” OR “dabigatran” OR “rivaroxaban” OR “apixaban” OR “edoxaban”	39,974
#4	#1 AND #2 AND #3	1,131
**Embase**		
#1	'atrial fibrillation' OR 'atrial flutter'	209,086
#2	'intracranial hemorrhage' OR 'intracranial bleeding' OR 'intracerebral hemorrhage' OR 'hemorrhagic stroke' OR 'ICH'	63,895
#3	'oral anticoagulant' OR 'vitamin K antagonist' OR 'VKA' OR ‘warfarin’ OR 'non-vitamin K antagonist oral anticoagulant' OR 'direct oral anticoagulant' OR 'novel oral anticoagulant' OR 'NOAC' OR 'DOAC' OR 'dabigatran' OR 'rivaroxaban' OR 'apixaban' OR 'edoxaban'	154,129
#4	#1 AND #2 AND #3	2,659

Supplemental Table 3. Excluding studies with reasons in this meta-analysis

	Study items
NO.1	Sample size was less than 100 (n=5)
1	Abrantes CS, Pintalhao M, Tavares S, Fonseca L, Chaves PC. Anticoagulation after intracerebral hemorrhage in patients with atrial fibrillation: between Scylla and Charybdis. NEUROL SCI. 2021.
2	Vidal-Jordana A, Barroeta-Espar I, Sainz PM, Mateo J, Delgado-Mederos R, Marti-Fabregas J. [Intracerebral hemorrhage in anticoagulated patients: what do we do afterwards?]. NEUROLOGIA. 2012;27(3):136-142.
3	Claassen DO, Kazemi N, Zubkov AY, Wijdicks EF, Rabinstein AA. Restarting anticoagulation therapy after warfarin-associated intracerebral hemorrhage. Arch Neurol. 2008;65(10):1313-1318.
4	Viswanathan A, Rakich SM, Engel C, Snider R, Rosand J, Greenberg SM, Smith EE. Antiplatelet use after intracerebral hemorrhage. NEUROLOGY. 2006;66(2):206-209.
5	De Vleeschouwer S, Van Calenbergh F, van Loon J, Nuttin B, Goffin J, Plets C. Risk analysis of thrombo-embolic and recurrent bleeding events in the management of intracranial haemorrhage due to oral anticoagulation. ACTA CHIR BELG. 2005;105(3):268-274.
NO.2	Studies did not reported adjusted or weighted HRs (n=6)
1	Sadighi A, Beckett L, DiCristina H, Wagner T, Wright K, Capone K, Monczewski M, Kester M, Bourdages G, Griessenauer C, Zand R. Long-term outcome of resuming anticoagulation after anticoagulation-associated intracerebral hemorrhage. eNeurologicalSci. 2020:100222.
2	Roquer J, Vivanco-Hidalgo RM, Prats-Sánchez LL, Martínez-Domeño A, Guisado-Alonso D, Cuadrado-Godia E, Giralt Steinhauer E, Jiménez-Conde J, Rodríguez-Campello A, Martí-Fàbregas J, Ois A. Interaction of atrial fibrillation and antithrombotics on outcome in intracerebral hemorrhage. NEUROLOGY. 2019;93(19):e1820-e1829.
3	Janumpally R, Ambartsumyan AS, Duan L, Gharibian D, Sangha N. Clinical outcomes of direct oral anticoagulants versus warfarin in patients with atrial fibrillation who have had an intracerebral hemorrhage. STROKE. 2018;49.
4	Pennlert J, Overholser R, Asplund K, Carlberg B, Van Rompaye B, Wiklund P, Eriksson M. Optimal Timing of Anticoagulant Treatment After Intracerebral Hemorrhage in Patients With Atrial Fibrillation. STROKE. 2017;48(2):314-320.
5	Yung D, Kapral MK, Asllani E, Fang J, Lee DS. Reinitiation of anticoagulation after warfarin-associated intracranial hemorrhage and mortality risk: the Best Practice for Reinitiating Anticoagulation Therapy After Intracranial Bleeding (BRAIN) study. CAN J CARDIOL.

	2012;28(1):33-39.
6	Majeed A, Kim Y, Roberts RS, Holmström M, Schulman S. Optimal Timing of Resumption of Warfarin After Intracranial Hemorrhage. STROKE. 2010;41(12):2860-2866.
**NO.3**	**Studies focused on a mixed population, and AF subgroup was not separately analyzed (n=3)**
1	Poli D, Antonucci E, Vignini E, Martinese L, Testa S, Simioni P, Pengo V, Pignatelli P, Falanga A, Masciocco L, Barcellona D, Ciampa A, Chiarugi P, Paparo C, Ageno W, Palareti G. Anticoagulation resumption after intracranial hemorrhage in patients treated with VKA and DOACs. EUR J INTERN MED. 2020;80:73-77.
2	Billings JD, Khan AD, McVicker JH, Schroeppel TJ. Newer and Better? Comparing Direct Oral Anticoagulants to Warfarin in Patients With Traumatic Intracranial Hemorrhage. Am Surg. 2020;86(9):1062-1066.
3	Ottosen TP, Grijota M, Hansen ML, Brandes A, Damgaard D, Husted SE, Johnsen SP. Use of Antithrombotic Therapy and Long-Term Clinical Outcome Among Patients Surviving Intracerebral Hemorrhage. STROKE. 2016;47(7):1837-1843.
**NO.4**	**Studies did not reported the studied outcomes (n=4)**
1	Stanton RJ, Eckman MH, Woo D, Moomaw CJ, Haverbusch M, Flaherty ML, Kleindorfer DO. Ischemic Stroke and Bleeding: Clinical Benefit of Anticoagulation in Atrial Fibrillation After Intracerebral Hemorrhage. STROKE. 2020;51(3):808-814.
2	Kato Y, Hayashi T, Suzuki K, Maruyama H, Kikkawa Y, Kurita H, Takao M. Resumption of Direct Oral Anticoagulants in Patients with Acute Spontaneous Intracerebral Hemorrhage. Journal of Stroke and Cerebrovascular Diseases. 2019;28(10):104292.
3	Vestergaard AS, Skjoth F, Lip GY, Larsen TB. Effect of Anticoagulation on Hospitalization Costs After Intracranial Hemorrhage in Atrial Fibrillation: A Registry Study. STROKE. 2016;47(4):979-985.
4	Pennlert J, Asplund K, Carlberg B, Wiklund P, Wisten A, Åsberg S, Eriksson M. Antithrombotic Treatment Following Intracerebral Hemorrhage in Patients With and Without Atrial Fibrillation. STROKE. 2015;46(8):2094-2099.
**NO.5**	**Studies focused on AF patients with non-ICH bleeding (n=4)**
1	Yokoyama M, Mizuma A, Terao T, Tanaka F, Nishiyama K, Hasegawa Y, Nagata E, Nogawa S, Kobayashi H, Yanagimachi N, Okazaki T, Kitagawa K, Takizawa S. Effectiveness of Nonvitamin K Antagonist Oral Anticoagulants and Warfarin for Preventing Further Cerebral Microbleeds in Acute Ischemic Stroke Patients with Nonvalvular Atrial Fibrillation and At Least One Microbleed: CMB-NOW Multisite Pilot Trial. Journal of Stroke and Cerebrovascular Diseases. 2019;28(7):1918-1925.

2	Adeboyeje G, Sylwestrzak G, Barron JJ, White J, Rosenberg A, Abarca J, Crawford G, Redberg R. Major Bleeding Risk During Anticoagulation with Warfarin, Dabigatran, Apixaban, or Rivaroxaban in Patients with Nonvalvular Atrial Fibrillation. J Manag Care Spec Pharm. 2017;23(9):968-978.
3	Lau WCY, Li X, Wong ICK, Man KKC, Lip GYH, Leung WK, Siu CW, Chan EW. Bleeding-related hospital admissions and 30-day readmissions in patients with non-valvular atrial fibrillation treated with dabigatran versus warfarin. J THROMB HAEMOST. 2017;15(10):1923-1933.
4	Hernandez I, Zhang Y, Brooks MM, Chin PKL, Saba S. Anticoagulation Use and Clinical Outcomes After Major Bleeding on Dabigatran or Warfarin in Atrial Fibrillation. STROKE. 2017;48(1):159-166.

Supplemental Table 4. Classifications of ICH and diagnostic methods in the included studies

Included studies	Classification of ICH				Diagnostic methods		
	Traumatic	OAC-related	Spontaneous	No classification	Brain MRI	Brain CT	Inpatients Medical Record
Komen-2021	×	×	×	√	√	√	√
Lee-2020	×	×	√	×	√	√	√
Tsai-2020	×	×	×	√	√	√	√
Newman-2020	×	×	×	√	√	√	√
Nielsen-2019	×	×	√	×	√	√	√
Perreault-2019	×	×	×	√	√	√	√
Nielsen-2017	√	√	√	×	√	√	√
Chao-2016	√	√	√	×	√	√	√
Park-2016	×	×	×	√	√	√	√
Nielsen-2015	×	×	×	√	√	√	√
Kuramatsu-2015	×	√	√	×	√	√	√
Lin-2022	×	×	×	√	√	√	√
Lewis-2021	×	×	√	×	√	√	√
Schreuder-2021	×	×	√	×	√	√	√

ICH= intracranial hemorrhage ; MRI= magnetic resonance imaging; CT= computerized tomography; “√” = study included this ICH classification or ICH was diagnosed by this method; “×” = study did not include this ICH classification nor ICH was diagnosed by this method.

Supplemental Table 5. Statistical methods and weighted/adjusted risk factors in the included studies

Included studies	Effect estimates	Statistical methods	Weighted or adjusted risk factors
Lewis-2021	Hazard ratio	Primary outcome: Cox regression model	Primary outcome: the time since ICH onset; ICH types<br><br>Secondary outcome: major vascular event, stroke, and stroke or vascular death
		Secondary outcome: Cox regression model	
Schreuder-2021	Hazard ratio	Primary outcome: Cox regression model	Primary outcome: age and ICH location<br><br>Secondary outcome: recurrent ICH, all major hemorrhagic, occlusive and vascular events
		Secondary outcome: Cox regression model	
Komen-2021	Adjusted Hazard ratio	Cox proportional hazards model	Age, sex, individual components of the Charlson Comorbidity Index, CHA2DS2-VASc score, modified HAS-BLED score, comorbidities (hypertension, diabetes mellitus, dyslipidemia, prior stroke, coronary heart disease, congestive heart failure, abnormal kidney function, abnormal liver function, anti-platelet medication), use of OACs
Lee-2020	Hazard ratio	Multivariable Cox proportional hazards regression models	Age, sex, CHA2DS2-VASc score, HAS-BLED score, Charlson comorbidity index, comorbidities (hypertension, diabetes mellitus, dyslipidemia, prior stroke, coronary heart disease, congestive heart failure, abnormal kidney function, abnormal liver function, anti-platelet medication), concomitant medication (aspirin, clopidogrel, dual antiplatelet, etc.)

Tsai-2020	Hazard ratio	Multivariable Cox proportional hazards regression analysis	Age, sex, comorbidities (hypertension, diabetes mellitus, dyslipidemia, prior stroke, coronary heart disease, congestive heart failure, abnormal kidney function, abnormal liver function, anti-platelet medication), use of antiplatelet drugs, and CHA2DS2-VASc score
Newman-2020	Hazard ratio	Cox proportional hazards models	Age, sex, race, comorbidities (hypertension, diabetes mellitus, dyslipidemia, prior stroke, coronary heart disease, congestive heart failure, abnormal kidney function, abnormal liver function, anti-platelet medication), ischemic stroke/TIA, TE, bleeding, CHADS2-VASc score, HAS-BLED score, and concomitant medication (aspirin, clopidogrel, dual antiplatelet, etc.)
Nielsen-2019	Risk ratio	Generalized linear model	Age, sex, stroke severity category, days since hospital discharge , length of hospital stay for the index intracerebral hemorrhage event, reduced renal function, alcohol consumption, smoking status, CHA2DS2 -VASc score, and aspirin treatment
Perreault-2019	Adjusted hazard ratios	Cox proportional hazard models	Age, sex, prior thromboembolism (stroke/TIA/SE) and major bleeding (except ICH), comorbidities (hypertension, diabetes mellitus, dyslipidemia, prior stroke, coronary heart disease, congestive heart failure, abnormal kidney function, abnormal liver function, anti-platelet medication), Score Charlson, CHA2DS2-VASc score, HAS-BLED score, length of stay, and concomitant medication (aspirin, clopidogrel, dual antiplatelet, etc.)

Nielsen-2017	Adjusted hazard ratios	Time-dependent Cox proportional hazards regression models	Age, sex, CHA2DS2-VASc score ,HAS-BLEDscore, prior previous thromboembolism, comorbidities (hypertension, diabetes mellitus, dyslipidemia, prior stroke, coronary heart disease, congestive heart failure, abnormal kidney function, abnormal liver function, anti-platelet medication), days in hospital from the index event and concomitant medication (aspirin, clopidogrel, dual antiplatelet, etc.)
Chao-2016	Adjusted hazard ratios	Cox regression analysis	Age, sex, CHA2DS2-VASc score, comorbidities (hypertension, diabetes mellitus, dyslipidemia, prior stroke, coronary heart disease, congestive heart failure, abnormal kidney function, abnormal liver function, anti-platelet medication), prior stroke/TIA, mean propensity score
Park-2016	Hazard ratio	Cox proportional hazards model	Age, sex,prior ischemic stroke, and previous warfarin medication
Nielsen-2015	Adjusted hazard ratio	Cox proportional hazard model	Age; sex; year of inclusion; time since last claimed OAC prescription before the incident ICH event; CHA2DS2-VASc score and HAS-BLED score
Kuramatsu-2015	Hazard ratio	Multivariable regression analysis	Age, sex, comorbidities (hypertension, diabetes mellitus, dyslipidemia, prior stroke, coronary heart disease, congestive heart failure, abnormal kidney function, abnormal liver function, anti-platelet medication), medication (aspirin, clopidogrel, dual antiplatelet, etc.), CHADS2 score, HAS-BLED score, initial imaging, time windows

Lin-2022	Hazard ratio	Cox proportional hazards model	Age, sex, prior comorbidities (congestive heart failure, hypertension, diabetes, coronary artery disease, myocardial infarction, peripheral artery disease, IS, transient ischemic attack, peripheral arterial occlusive disease, venous thromboembolism, pulmonary embolism, gastrointestinal bleeding, other bleeding events, liver disease, and renal disease), and medications (calcium channel blockers, renin-angiotensin system inhibitors, 3-hydroxy-3-methylglutaryl coenzyme A reductase inhibitors, non-steroidal anti-inflammatory drugs, class I and III anti-arrhythmic drugs, β blockers, digoxin, and proton pump inhibitors.

ICH= intracranial hemorrhage ; TIA= transient ischemic attack; SE= systemic embolism; CHA2DS2-VASc=congestive heart failure/left ventricular ejection fraction ≤ 40%, hypertension, age ≥ 75 years (2 points), diabetes mellitus, prior stroke/transient ischemic attack/thromboembolism (2 points), vascular disease, age 65-74 years, female sex; HAS-BLED=Hypertension, Abnormal liver/renal function, Stroke, Bleeding history or predisposition, Labile international normalized ratio, Elderly, Drugs/alcohol concomitantly.

Supplementary Table 6. Risk of bias assessment for randomized clinical trials

Contents for risk assessment	Assessment justification	Ratings
**Lewis-2021**		
Random sequence generation (selection bias)	A central, web-based, computerized randomization system applying a minimization algorithm randomly assigned participants (1:1) to initiate and stop anticoagulant treatment	Low risk
Allocation concealment (selection bias)	The unique study identification number was allocated to every participants who were administrated to oral anticoagulants	Low risk
Blinding of participants and personnel (performance bias)	Participants, clinicians and local investigators knew the treatment assignments, but participant identity, treatment allocation, and drug use was unknown to event adjudicators	Moderate risk
Blinding of outcome assessment (detection bias)	Reports of every outcome events were performed by internal assessor -- one medically trained clinical research fellow, and investigators rated dependence and quality of life was evaluated by modified Rankin Scale and EQ-5D-5L, respectively	Low risk
Incomplete outcome data (attrition bias)	Completeness of follow-up was performed by follow-up questionnaire at each planned interval after randomization	Low risk
Selective reporting (reporting bias)	NS	UNCLEAR
Other risk biases	NS	UNCLEAR

Schreuder-2021		
Random sequence generation (selection bias)	Participants were (1:1) randomly allocated by a central computerized randomization system to either apixaban and stop anticoagulant treatment	Low risk
Allocation concealment (selection bias)	Treatment assignment was known to participants, their treating physicians, and local investigators	High risk
Blinding of participants and personnel (performance bias)	Treatment assignment was known to participants, their treating physicians, and local investigators	High risk
Blinding of outcome assessment (detection bias)	The adjudication committee accessing all potential outcomes was not aware of patient identity, treatment allocation, and drugs used; two neurologists with neurovascular expertise (LJK and GJER) and a cardiologist (H M Nathoe) were composed of this committee	Low risk
Incomplete outcome data (attrition bias)	All participants were included in this study	Low risk
Selective reporting (reporting bias)	NS	UNCLEAR
Other risk biases	NS	UNCLEAR

EQ-5D-5L=five-level EuroQol five-dimensional questionnaire

.

Supplementary Table 7. Quality assessment for observational cohorts using the NOS tool

Included studies	Selection (0-4 points)				Comparability (0-2 points)		Outcome (0-3 points)			Total points
	Representativeness of Exposed Cohort	Selection of Non-Exposed Cohort	Ascertainment Of Exposure	Demonstration That Outcome of Interest Was Not Present at Start of Study	Adjust for the important Risk factors	Adjust for other risk factors	Assessment of outcome	Follow-up length	Loss to follow-up rate	
Komen-2021	1	1	1	1	1	1	1	0	1	8
Lee-2020	1	1	1	0	1	1	1	1	1	8
Tsai-2020	1	1	1	0	1	1	1	1	1	8
Newman-2020	1	1	1	0	1	1	1	1	1	8
Nielsen-2019	1	1	1	0	1	1	1	1	1	8
Perreault-2019	1	1	1	1	1	1	1	1	1	9
Nielsen-2017 [#]	1	1	1	1	1	1	1	1	1	9
Chao-2016	1	1	1	1	1	1	1	1	1	9
Park-2016	1	1	1	1	1	1	1	1	1	9
Nielsen-2015	1	1	1	1	1	1	1	1	1	9
Kuramatsu-2015	1	1	1	1	1	1	1	1	1	9
Lin-2022	1	1	1	1	1	1	1	1	1	9

[#] only used in the subgroup analysis of vitamin-K antagonists (VKAs) versus no VKAs. NOS=Newcastle-Ottawa Scale.

Supplementary Table 8. The publication bias assessed using the Egger's and Begg's tests

Outcomes	OACs versus no OACs			VKAs versus no VKAs			NOACs versus VKAs		
	Number of studies	Egger's (P-value)	Begg's (P-value)	Number of studies	Egger's (P-value)	Begg's (P-value)	Number of studies	Egger's (P-value)	Begg's (P-value)
Stroke or systemic embolism	6	0.052 *	0.707	4	0.036 *	0.089	-	-	-
Ischemic stroke	4	0.464	0.734	4	0.111	0.308	4	0.780	0.734
All-cause death	7	0.041 *	0.368	-	-	-	4	0.931	1.000
Intracranial hemorrhage	7	0.212	0.548	3	0.461	1.000	4	0.988	0.734
Major bleeding	5	0.674	0.806	-	-	-	-	-	-

* The results from the trim-and-fill analysis showed no trimming performed, and the corresponding pooled results were not changed.

Supplemental Table 9. Ongoing RCTs including patients with AF after ICH

Name	Number	Sample size	Included experimental group and control group
STATICH	NCT0318 6729	500 participants	Experimental group: with anticoagulant treatment<br>Control group: with no anticoagulant treatment
PRESTIGE-A F	NCT0399 6772	654 patients	Experimental group: with DOAC treatment<br>Control group: with no anticoagulant treatment
ENRICH-AF	NCT0395 0076	1200 patients	Experimental group: with edoxaban treatment<br>Control group: with no anticoagulant treatment
A3ICH	NCT0324 3175	300 patients	Experimental group: with apixaban treatment<br>Control group: with LAA occlusion treatment
ASPIRE	NCT0390 7046	700 patients	Experimental group: with apixaban treatment<br>Control group: with aspirin treatment

RCT=randomized clinical trial; STATICH= study of antithrombotic treatment after intracerebral hemorrhage; PRESTIGE-AF=prevention of stroke in intracerebral haemorrhage survivors with atrial fibrillation; ENRICH-AF=edoxaban for intracranial hemorrhage survivors with atrial fibrillation; A3ICH=avoiding anticoagulation after intracerebral haemorrhage; ASPIRE=anticoagulation in ICH survivors for stroke prevention and recovery; DOAC=direct oral anticoagulant; LAA=left atrial appendage

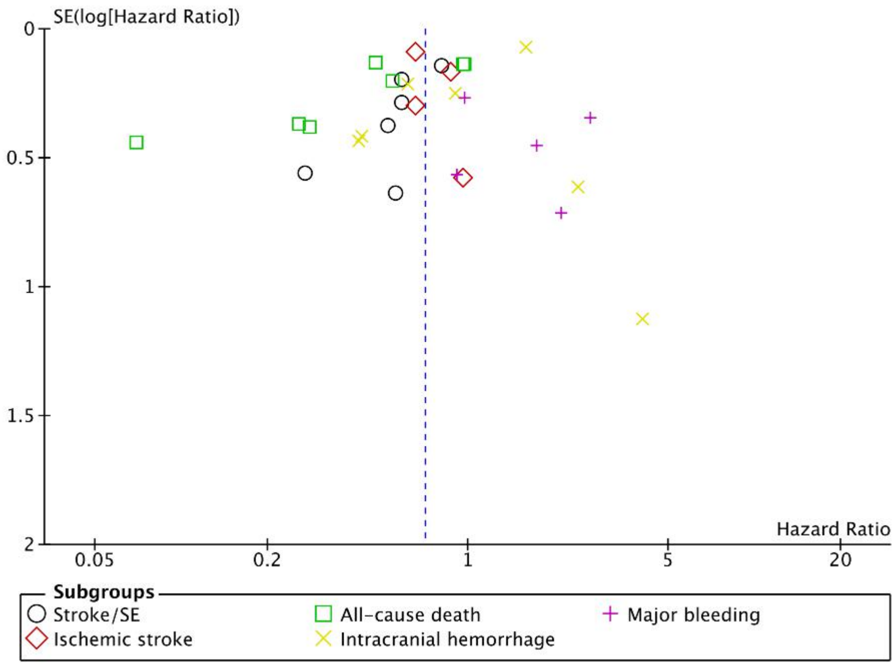

Supplementary Figure 1. Funnel plots for OACs versus no OACs in AF patients after ICH

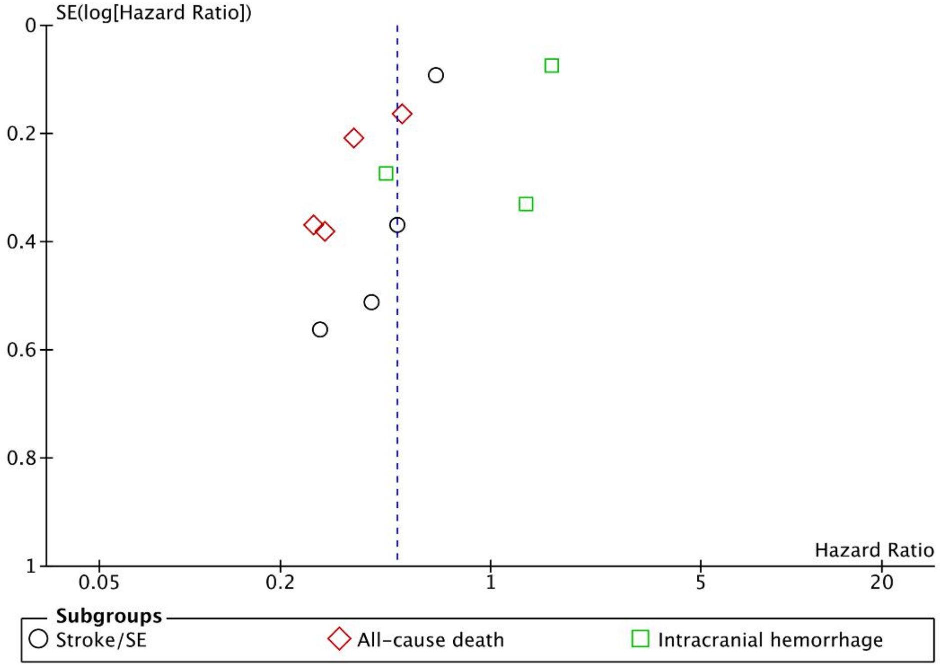

Supplementary Figure 2. Funnel plots for VKAs versus no VKAs in AF patients after ICH

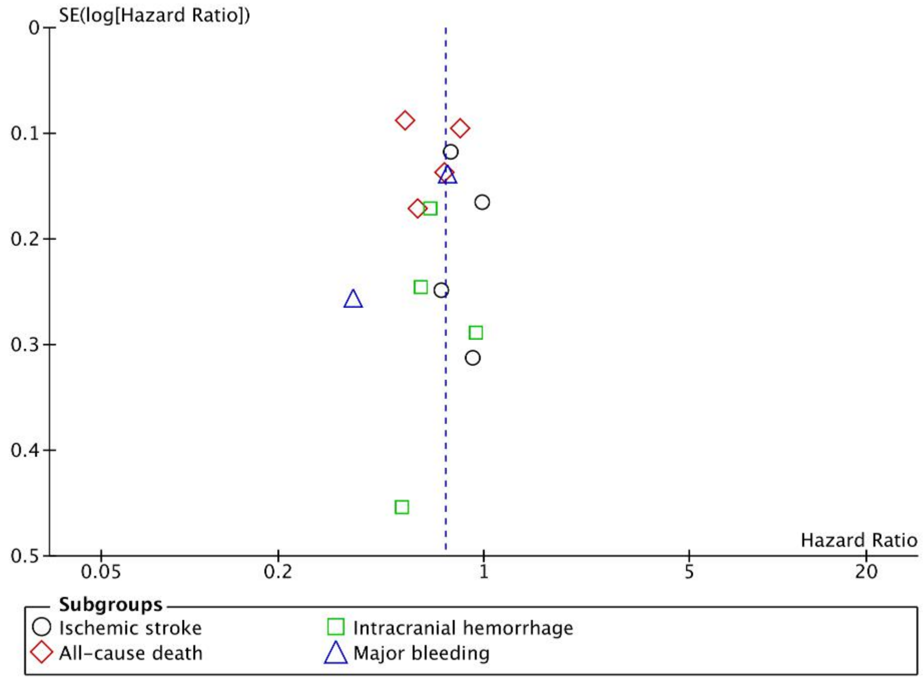

Supplementary Figure 3. Funnel plots for NOACs versus VKAs in AF patients after ICH
